# Improving immunization capacity in Ethiopia through continuous quality improvement interventions: a prospective quasi-experimental study

**DOI:** 10.1186/s40249-018-0502-8

**Published:** 2018-11-30

**Authors:** Tsegahun Manyazewal, Alemayehu Mekonnen, Tesfa Demelew, Semegnew Mengestu, Yusuf Abdu, Dereje Mammo, Workeabeba Abebe, Belay Haffa, Daniel Zenebe, Bogale Worku, Amir Aman, Setegn Tigabu

**Affiliations:** 1 Ethiopian Public Health Association, P.O. Box 7117, Addis Ababa, Ethiopia; 2 Ethiopian Medical Association, P.O. Box 2179, Addis Ababa, Ethiopia; 3 Ethiopian Pediatrics Society, P.O. Box 14205, Addis Ababa, Ethiopia; 4 Department of Pediatrics and Child Health, School of Medicine, College of Health Sciences, Addis Ababa University, P.O. Box 9086, Addis Ababa, Ethiopia; 5 Federal Ministry of Health, Government of Ethiopia, P.O. Box 1234, Addis Ababa, Ethiopia; 6 Center for Innovative Drug Development and Therapeutic Trials for Africa (CDT-Africa), College of Health Sciences, Addis Ababa University, P.O. Box 9086, Addis Ababa, Ethiopia

**Keywords:** Immunization, Continuous quality improvement, Vaccination, Expanded program on immunization, Ethiopia

## Abstract

**Background:**

Strong scientific evidence is needed to support low-income countries in building effective and sustainable immunization programs and proactively engaging in global vaccine development and implementation initiatives. This study aimed to implement and evaluate the effectiveness of system-wide continuous quality improvement (CQI) interventions to improve national immunization programme performance in Ethiopia.

**Methods:**

The study used a prospective, quasi-experimental design with an interrupted time-series analysis to collect data from 781 government health sectors (556 healthcare facilities, 196 district health offices, and 29 zonal health departments) selected from developing and emerging regions in Ethiopia. Procedures included baseline quality assessment of immunization programme and services using structured checklists; immunization systems strengthening using onsite technical support, training, and supportive supervision interventions in a Plan-Do-Check-Act cycle over 12 months; and collection and analysis of data at baseline and at the 6th and 12th month of interventions using statistical process control and the *t*-test. Outcome measures were the coverage of the vaccines pentavalent 3, measles, Bacillus Calmette–Guérin vaccine (BCG), Pneumococcal Conjugate Vaccine (PCV), as well as full vaccination status; while process measures were changes in human resources, planning, service delivery, logistics and supply, documentation, coordination and collaboration, and monitoring and evaluation. Analysis and interpretation of data adhered to SQUIRE 2.0 guidelines.

**Results:**

Prior to the interventions, vaccination coverage was low and all seven process indicators had an aggregate score of below 50%, with significant differences in performance at healthcare facility level between developing and emerging regions (*P* = 0.0001). Following the interventions, vaccination coverage improved significantly from 63.6% at baseline to 79.3% for pentavalent (*P* = 0.0001), 62.5 to 72.8% for measles (*P* = 0.009), 62.4 to 73.5% for BCG (*P* = 0.0001), 65.3 to 81.0% for PCV (*P* = 0.02), and insignificantly from 56.2 to 74.2% for full vaccination. All seven process indicators scored above 75% in all regions, with no significant differences found in performance between developing and emerging regions.

**Conclusions:**

The CQI interventions improved immunization capacity and vaccination coverage in Ethiopia, where the unstable transmission patterns and intensity of infectious diseases necessitate for a state of readiness of the health system at all times. The approach was found to empower zone, district, and facility-level health sectors to exercise accountability and share ownership of immunization outcomes. While universal approaches can improve routine immunization, local innovative interventions that target local problems and dynamics are also necessary to achieve optimal coverage.

## Background

Vaccination has greatly lowered the burden of infectious diseases since the start of the Expanded Program on Immunization (EPI) by the World Health Organization (WHO) in 1974, reducing mortality, morbidity, and saving resources [1–4]. Vaccine innovations have been cultivated to tackle emerging and re-emerging diseases [5, 6] and combat antimicrobial resistance [7]. However, regardless of the rapidly changing immunization landscape, immunization programmes in resource limited countries still lag in bringing universal access of all relevant vaccines to all at-risk groups [8–10].

Ethiopia is among the countries adversely experiencing repeated epidemics and outbreaks of vaccine-preventable diseases, despite strong government-led efforts to combat this. A large proportion of vulnerable infants and children in Ethiopia are facing vaccine-preventable deaths, especially in communities that are hard-to-reach, poor, and sparsely populated. The deaths are mainly due to diarrheal diseases (18%) and pneumonia (18%) [11]. These deaths are intrinsically linked to a lack of timely, evidence-based interventions, which are more complex to deal with. Scientific literature in Ethiopia has affirmed that regional disparities [11, 12], administrative barriers [13], poor reporting [14], and a lack of skilled human resources [15] are serious challenges for the nation’s immunization programme. In 2013, in light of such challenges, the Ethiopian Federal Ministry of Health (FMoH) initiated a national routine immunization improvement plan, which was a two-year (2014–2015) plan intended to improve national routine immunization programme performance and ensure equity of coverage among different populations [11]. In 2015, the FMoH developed a comprehensive multi-year plan of action, which covers the fiscal years 2016–2020, to expand the former plan and standardize entry of new vaccines into the country [16]. Ethiopia’s individual and collective efforts with partners have resulted in some positives for the overall immunization system, including improved immunization coverage and accessibility of some new vaccines. However, these efforts have not been integrated and cultivated by locally appropriate quality improvement systems capable of achieving targets in the national immunization plan. National immunization activities are monitored using casual supervisory visits with less autonomy and opportunities given to district health bureaus to resolve bottlenecks onsite. The current national immunization plan requires new intervention mechanisms that would nurture the capacity and responsiveness of lower-level governments to own immunization programmes and mobilize the community to share responsibilities.

This study hypothesized that employing continuous quality improvement (CQI) interventions to assess, improve, and continuously follow-up immunization programme and services is an effective and sustainable approach to achieve Ethiopia’s national immunization improvement plan. CQI is an iterative procedure of planning to improve a process, plan implementation, analyse and compare results, and take corrective actions [17]. Today’s healthcare organizations are rapidly expanding CQI interventions worldwide to foster quality and sustainability of healthcare programmes and services [18–21].

**Fig. 1:**
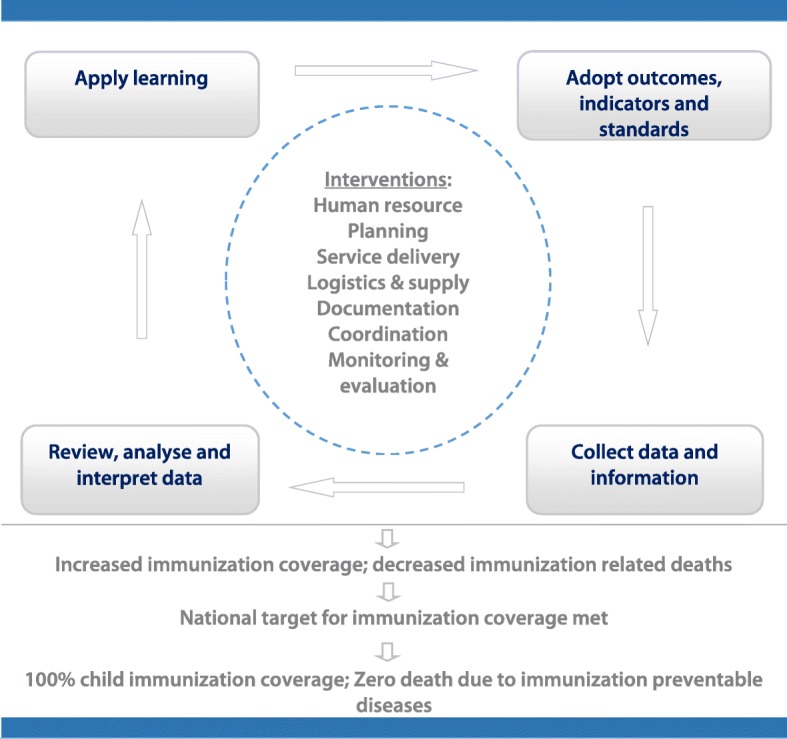
Conceptual framework of the study design based on the complete system of CQI in a PDCA cycle. CQI: Continuous quality improvement; PDCA: Plan-Do-Check-Act

The conceptual framework of the study is summarized in Fig. 1. The framework is designed based on the complete system of CQI in a Plan-Do-Check-Act (PDCA) cycle and the major areas of improvement of the Ethiopian routine immunization improvement plan [11]. The PDCA is a repetitive four-step management model for the control and continual improvement of processes and services [22]. Thus, this study aimed to implement and evaluate the effectiveness of system-wide CQI interventions to improve national immunization programme performance in Ethiopia.

## Methods

### Context

The study was performed in Ethiopia, where cases of vaccine-preventable diseases continue to exist [23]. The country is a federal state comprising nine regional states and two chartered cities. According to their level of economic and social development, the nine regional states are classified as developing and emerging regions. The former includes Amhara, Harari, Oromia, the Southern Nations, Nationalities and Peoples’ Region (SNNPR), and Tigray, while the later includes Afar, Somali, Benishangul-Gumuz, and Gambella [24]. Compared with the developing regions, the emerging regions have a predominance of pastoral and agropastoral livelihoods with limited access to infrastructure and public services. The emerging regions lag behind in development, poverty reduction, and private investment [25]. The overall 11 administrative states are subdivided into 68 administrative zones and 817 districts, and the health system is structured accordingly in a top-down hierarchy [26].

In response to the FMoH’s call for collaboration on the national routine immunization improvement plan, a consortium of three civil society organizations, namely, the Ethiopian Public Health Association, Ethiopian Medical Association, and Ethiopian Pediatrics Society, has worked since 2014 with the FMoH and Gavi, the Vaccine Alliance to design an innovative project on immunization. The goal of the consortium’s project, entitled “Integrated approach to Empower Healthcare facilities on Immunization (IEHI)” was to ensure implementation and management of the best immunization services by public healthcare workers and their coordinators, which would have a sustainable and strong impact on immunization capacity in Ethiopia. The current study encloses the IEHI project, reinforcing scientific and evidence-based practices to improve immunization programmes in the country.

The target institutions of the study were zonal health departments, district health offices, and healthcare facilities in Ethiopia. The selection of the study sites was based on stratified multi-stage sampling. The nine regional states were stratified into two groups based on their formal level of economic and social development: developing regions (*n* = 5) and emerging regions (*n* = 4). Three regions were randomly selected from each group, making a total of six regions. From each of the six regions, all zonal health departments, district health offices, and local healthcare facilities were identified and checked individually adhering to certain criteria. The criteria included availability of immunization programme or services at the time of site selection; willingness of the site to enrol in the study and carry out ongoing interventions; and the site not already receiving technical support from other non-governmental EPI implementing partners.

The target participants of the study were EPI experts who manage and coordinate immunization programmes at zone and district levels, and healthcare workers who provide immunization services at healthcare facility and community levels. A participant was considered eligible if he/she was a full-time employee at the study site responsible for EPI programmes or services and was willing to give consent to participate. With this, 225 EPI experts who manage and coordinate immunization programmes at zone and district levels, and 556 healthcare workers who provide immunization services at healthcare facility and community levels were involved. Fifteen independent EPI consultants and three supervisors provided onsite technical support and supportive supervision (SS) visits. The independent consultants had a minimum of a master’s degree in a health-related field of study and ample experience in managing, supervising, and implementing immunization programmes and services. The consultants were trained for five days by the investigators before being involved in the study. The target beneficiaries were all children in the immunization age group in the six selected regions.

### Interventions

The study used a prospective, quasi-experimental design with an interrupted time-series applied in two phases. Phase I involved conducting a baseline assessment of study sites to serve as a benchmark for interventions. The baseline assessment was conducted over three months from October to December 2015 through onsite observations, preliminary interviews, and a review of facility data. The observations comprised inspecting availability and adequacy of immunization-related supplies, equipment, and immunization room setup. The data review involved assessing immunization-related plans, reports, relevant documents, communications, and monitoring and evaluation records. The interview segment involved face-to-face interviews with EPI experts and immunization service provides in order to investigate local views and enhance the study teams’ understanding of how immunization programmes and services are actually delivered, monitored, evaluated, and reported. The initial data were collected using three structured CQI checklists designed to extrapolate the current immunization issues and identify opportunities for improvement in line with the targets of Ethiopia’s national routine immunization improvement plan. The tools were designed independently for healthcare facilities, district health offices, and zonal health departments. Cronbach’s alpha was measured to confirm internal consistency of the data tools. Independent EPI consultants collected the data. The structure and contents of the data collection tools are explained below.

Phase II involved implementing CQI interventions in a health systems strengthening package, which included onsite technical support, training, and SS in a PDCA cycle based on pertinent needs identified in the baseline assessment and taking into account indicators of the national immunization improvement plan. Each site was informed of the baseline findings and discussions were held with the study site’s head, immunization focal person, maternal and child health focal person, and immunization service providers, as they were available. Then, each site was informed to develop plans to fill gaps identified in the baseline assessment. Follow-up assignments of developing long- and short-term plans were given at each site to ensure gaps identified in the baseline assessment were filled.

The training package was designed as a result of the baseline report, which indicated a high shortage of skilled workforce in immunization programmes and services. The target was for each site to have at least two staff trained on immunization to have the capacity to effectively manage the immunization programme or deliver immunization services. Therefore, a total of 513 EPI managers and coordinators at study zones and districts received a 10-day immunization for mid-level managers (MLM) training. The MLM curriculum was a readily available course designed by the WHO and approved by the FMoH [27]. The WHO designed the curriculum to help mid-level immunization managers effectively deliver vaccines to their communities. The standard course modules are: cold chain, vaccines, and safe-injection equipment management; partnering with the community; immunization safety; supportive supervision; monitoring the immunization system; making a comprehensive annual national plan; the EPI coverage survey; and making disease surveillance work. Each MLM module is organized around a series of steps in which technical information is provided, followed by learning activities.

Similarly, a total of 2236 healthcare providers involved in immunization services at healthcare facility and community levels received a five-day Immunization in Practice (IIP) training using the WHO’s IIP curriculum that was approved by the FMoH [28]. The standard IIP curriculum has ten modules and was designed for healthcare workers who deliver immunization services to children and women. The course modules are: EPI target diseases; EPI vaccines; the vaccine cold chain; ensuring safe injections; organizing immunization sessions; registering and assessing clients; preparing vaccines; giving immunizations; communicating with parents and involving communities; and monitoring immunization coverage.

Following the training, the 15 independent EPI consultants provided onsite technical support on a quarterly basis backed by frequent technical consultation phone calls. The technical support was given in all aspects of human resources, planning, service delivery, logistics and supply, documentation, coordination and collaboration, and monitoring and evaluation. The consultants worked closely with site immunization focal persons to solve bottlenecks and maximize performance and uninterrupted immunization services. Three supervisors regularly rechecked and monitored activities of the consultants to ensure the quality of interventions. Using the CQI checklists, immunization staff and the site’s head met monthly to discuss tested changes and to plan new tests of changes. These interventions lasted 12 months from January to December 2016.

### Measures

The effectiveness of the CQI interventions was measured in terms of ‘process’ and ‘outcome’ indicators. The process was measured based on seven parameters at healthcare facility level and six parameters at district and zone levels: human resources, planning, logistics and supply, service delivery (only for healthcare facilities), documentation, coordination and collaboration, and monitoring and evaluation. The parameters were derived from core indicators that the Ethiopian national immunization improvement plan targets to achieve. The seven parameters and their detailed components and scoring are described in Table 1.

**Table 1:** Process measures, their components and scores

Process measures	Components	Scores (point)
1. Human resources	1.1. Proportion of zone, district, and healthcare facilities (HFs) with a designated immunization focal person 1.2. Proportion of zone and district with at least 2 MLM-trained experts 1.3. Proportion of HFs with at least 2 IIP-trained experts 1.4. Proportion of zone, district, and HFs with a designated cold chain officer 1.5. Proportion of zone and district with an expert/technician on mid-level cold chain management 1.6. Proportion of HFs with an expert on user/preventive cold chain	4 at zone, district, and HF
2. Planning	2.1. Proportion of zone, district, and HFs with Reaching Every Community (REC) microplan 2.2. Proportion of HFs with outreach/mobile service delivery plan 2.3. Proportion of HFs with outreach/mobile REC microplan (if available), posted on the wall	3 at HF,2 at zone and district
3. Logistics and supply	3.1. Proportion of zone, district, and HFs with an adequate fridge-tag 2 3.2. Proportion of zone, district, and HFs with refrigerator spare parts 3.3. Proportion of zone, district, and HFs with vaccine request and report forms 3.4. Proportion of zone, district, and HFs with EPI vaccine and injection materials stock ledger book 3.5. Proportion of zone, district, and HFs with inventory documents 3.6. Proportion of HFs with adequate cold box 3.7. Proportion of HFs with adequate vaccine carrier with foam pads 3.8. Proportion of HFs with adequate vaccine storage capacity 3.9. Proportion of HFs with adequate water/ice packs 3.10. Proportion of zone, district, and HFs with no stock running out in the last 3 months 3.11. Proportion of zone, district, and HFs with refrigerator temperature monitored twice daily using fridge-tag 2 3.12. Proportion of zone, district, and HFs with no expired vaccines in refrigerator, or acceptable Vaccine Vial Monitor 3.13. Proportion of zone, district, and HFs with procedures and actions for vaccines exposed to extreme temperatures	13 at HF,9 at zone and district
4. Service Delivery	4.1. Proportion of HFs with immunization services available 4.2. Proportion of HFs with regular static immunization services delivered 4.3. Proportion of HFs with adequate outreach sites 4.4. Proportion of HFs with catchment area mapped for immunization 4.5. Proportion of HFs with separate and adequate rooms to deliver immunization services and store supplies 4.6. Proportion of HFs with all planned outreach sessions conducted 4.7. Proportion of HFs providing health education on immunization 4.8. Proportion of HFs with immunization services available under all their catchment health posts	8 at HF, 0 at zone and district
5. Documentation	5.1. Proportion of zone, district, and HFs with electronic database for monitoring immunization and staff training 5.2. Proportion of zone, district, and HFs with standard operating procedures on immunization 5.3. Proportion of HFs with reports received from all catchment health posts and the reports are well documented 5.4. Proportion of HFs with tally sheets appropriately documented 5.5. Proportion of HFs with defaulter tracking list 5.6. Proportion of HFs with standard case definition for neonatal tetanus, measles, acute flaccid paralysis (AFP) 5.7. Proportion of HFs with an up-to-date under-one register 5.8. Proportion of zone, district, and HFs with up-to-date national immunization guidelines	8 at HF, 3 at zone and district
6. Coordination and collaboration	6.1. Proportion of zone with established partners’ forum 6.2. Proportion of zone with partners’ forum meeting conducted as planned 6.3. Proportion of zone and district with EPI programme network 6.4. Proportion of district with established technical working group 6.5. Proportion of district with a technical working group meeting conducted in the last quarter 6.6. Proportion of HFs conducting a catchment area meeting in the last quarter 6.7. Proportion of HFs with established inter-facility linkage on immunization services	4 at zone and district, 2 at HF
7. Monitoring and evaluation	7.1. Proportion of zone, district, and HFs posting updated EPI monitoring chart 7.2. Proportion of HFs with vaccine logs 7.3. Proportion of HFs with vaccine and injection materials stock record book 7.4. Proportion of zone, district, and HFs conducting an internal review meeting on EPI in the last quarter 7.5. Proportion of zone, district, and HFs having a SS visit by government or partners in the last quarter 7.6. Proportion of zone, district, and HFs having a data quality check by the government or partners in the last quarter 7.7. Proportion of zone, district, and HFs with timeliness of monthly reports 7.8. Proportion of zone, district, and HFs with completeness of monthly reports 7.9. Proportion of zone, district, and HFs with a functional performance monitoring team 7.10. Proportion of zone and district with monitored vaccine wastage 7.11. Proportion of HFs conducting SS of health posts in the last three months 7.12. Proportion of HFs supervising community immunization programme in the last three months	11 at HF, 8 at zone and district

*EPI*: Expanded Program on Immunization; *HF*: Healthcare facility; *MLM*: Mid-level managers; *SS*: Supportive supervision

The outcome measures were represented by the coverage of five vaccinations: pentavalent 3, measles, Bacillus Calmette–Guérin (BCG), Pneumococcal Conjugate Vaccine (PCV), and full vaccination (fully immunized). The figures of baseline vaccination coverage of catchments were collected from immunization registers available at each zonal health department, district health office, and healthcare facility. Such data were captured every six months during CQI interventions to compare with the baseline and analyse changes due to interventions.

### Analysis

Data were entered into EPI Info™ version 3.5.1 (Centers for Diseases Control and Prevention, Atlanta, US) and transferred to Statistical Package for the Social Sciences (SPSS) version 22 (IBM Corp., Armonk, NY, US) for analysis. Analysis and interpretation of data adhered to SQUIRE 2.0 (Standards for Quality Improvement Reporting Excellence) guidelines [29] to ensure quality of data and methodology. Process measures were calculated numerically, where each element in the seven parameters had a value of one for a positive outcome or a value of zero for a negative or null outcome. Aggregating all scores of the seven process measures produced a composite variable score to indicate a study site’s status of immunization programme or services. The aggregate score of a study site was then changed to a percentage and classified: a score of below 50% was considered ‘low’, a score ranging between 50 and 75% was ‘fair’, and a score of above 75% was ‘good’.

Descriptive characteristics were summarized using counts, means, and proportions. A region’s cumulative score was calculated as the average score of the seven process measures of all zonal health departments, district health offices, and healthcare facilities in the region. A cumulative score of a developing or emerging region was calculated as the percentage mean score of the seven process measures of all zonal health departments, district health offices, and healthcare facilities in the developing or emerging regions. Descriptive characteristics of outcome measures followed a similar method but using percentages of vaccination coverage. Data were analysed numerically using statistical process control to determine changes due to interventions. A *t*-test analysis was carried out to describe whether there were changes in the coverage of outcome measures through time. The *t*-test for equality of means was also employed to check if there were changes in vaccinations at baseline and at 6th and 12th month of CQI interventions. The *P* value of < 0.05 was considered statistically significant.

### Ethical considerations

The study protocol was approved by the Internal Scientific and Ethical Review Committee of the Ethiopian Public Health Association. Permission to undertake the study was obtained from the FMoH and the Afar, Amhara, Gambella, Oromia, SNNPR, and Somali regional health bureaus. Privacies of staff involved were sufficiently protected.

## Results

**Fig. 2:**
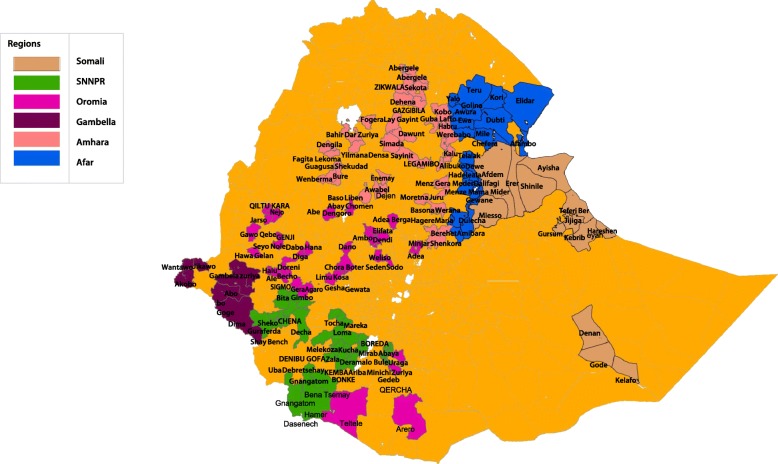
Map of Ethiopia showing study sites

The study included 783 government health sectors, comprising 558 local healthcare facilities, 196 district health offices, and 29 zonal health departments; this covers 64.5% of zonal and 36% of district health bureaus in the study regions. All sites were willing to participate. Figure 2 is a map of Ethiopia showing the various districts included in the study. Table 2 summarizes the type and distribution of study sites.

**Table 2:** Type and distribution of study sites

Regions	Zonal health department (f)	District health office (f)	Healthcare facility (f)	Total (f)
Developing regions				
Amhara	8	42	146	196
Oromia	10	43	155	208
SNNPR	8	44	120	172
Emerging regions				
Afar	NA	26	56	82
Gambella	3	13	29	45
Somali	NA	28	50	78
Total	29	196	556	781

*NA*: Not available; *SNNPR*: Southern nations, nationalities and Peoples’ Region

### Results of process measures

The process measures were changes in human resources, planning, service delivery, logistics and supply, documentation, coordination and collaboration, and monitoring and evaluation.

In terms of human resources, there were immunization focal persons in 96.6% of the zonal health departments, 90.3% of the district health offices, and 92.4% of the healthcare facilities at the time of baseline assessment, which was considered excellent. This figure was consistent at the 6th and 12th month of interventions at the zonal level, and it improved to around 98% at both the district and healthcare facility levels. The focal persons had the role of managing and coordinating immunization programmes and services at each level. In contrast, 82.8% of zonal health departments, 91.8% of district health offices, and 83.1% of healthcare facilities lacked the required number of experts who have undergone the MLM or IIP training (see Table 3). The percentage of experts trained in MLM increased from 17.2 to 82.1% at the zonal level and from 8.2 to 74.7% at the district level, and experts trained in IIP increased from 16.9 to 81.1% at the healthcare facility level after 12 months of interventions. This was due to training interventions being delivered to 513 MLM and 2236 IIP training participants. Consultations given to heads of study sites as part of onsite technical support resulted in a higher number of cold chain officers at each level (see Table 3). The average number of experts assigned for immunization at the zonal level was two at baseline, which was improved to three at the 6th month and four at the 12th month of interventions, with much improvement observed in Gambella, Oromia, and SNNPR. Similar improvements were recorded at the district level, except for Gambella, and at the healthcare facility level, except for Amhara.

**Table 3:** Percentage scores of process measures at baseline, and at the 6th and 12th month of interventions

Process measures	Baseline (%)			After 6 months (%)			After 12 months (%)		
	Zone	Dist.	HF	Zone	Dist.	HF	Zone	Dist.	HF
1. Human resources									
1.1. A designated immunization focal person	96.6	90.3	92.4	96.6	98.3	98.0	96.4	97.9	98.0
1.2. At least 2 MLM-trained experts	17.2	8.2	NA	86.2	61.4	NA	82.1	74.7	NA
1.3. At least 2 IIP-trained experts	NA	NA	16.9	NA	NA	85.4	NA	NA	81.1
1.4. A designated cold chain officer	58.6	43.9	32.1	79	70	43	82.1	67.4	51.6
1.5. An expert/technician on mid-level cold chain management	58.6	87.1	NA	79	62.4	NA	92.9	68.9	NA
1.6. An expert on user/preventive cold chain	NA	NA	35.2	NA	NA	30.7	NA	NA	39.3
2. Planning									
2.1. REC microplan 2.2. REC microplan (if available), posted on the wall	34.5 20.0	45.9 15.8	27.6 23.3	58.6 27.6	73.3 56.7	55.8 65.1	71.4 42.9	72.6 62.6	59.6 69.7
2.3. Outreach/mobile service delivery plan	NA	NA	29.7	NA	NA	57.1	NA	NA	60.0
3. Logistics and supply									
3.1. Adequate fridge-tag 2	75.9	71.9	72.8	82.8	78.0	78.6	75.0	72.1	82.2
3.2. Refrigerator spare parts	44.8	23.0	16.5	41.4	89.3	32.3	39.3	40.5	39.9
3.3. Vaccine request and report forms	79.3	65.8	47.5	82.8	89.3	65.5	82.1	78.4	71.6
3.4. EPI vaccine and injection materials stock ledger book	72.4	64.8	70.8	82.8	82.4	81.4	75.0	69.5	91.9
3.5. Inventory documents	51.7	34.7	21.9	58.6	65.6	42.4	67.9	57.4	44.1
3.6. Adequate cold box	NA	NA	62.5	NA	NA	82.1	NA	NA	91.6
3.7. Adequate vaccine carrier with foam pads	NA	NA	70.2	NA	NA	76.1	NA	NA	79.9
3.8. Adequate vaccine storage capacity	NA	NA	82.2	NA	NA	87.9	NA	NA	94.0
3.9. Adequate water/ice packs	NA	NA	77.8	NA	NA	91.2	NA	NA	95.1
3.10. No stock running out in the last 3 months	41.4	52.0	71.3	62.1	78.1	78.2	39.3	58.4	67.3
3.11. Refrigerator temperature monitored twice daily using fridge-tag 2	72.4	56.6	58.1	82.8	80.5	73.9	82.1	77.9	79.7
3.12. No expired/VVM beyond discard point vaccine in refrigerator	72.4	66.3	70.8	96.3	89.6	89.4	82.1	77.9	90.1
3.13. Procedures and actions for vaccines exposed to extreme temperatures	51.7	52.9	54.4	65.5	77.3	63.4	75.0	56.8	66.5
4. Service delivery									
4.1. Immunization services available	NA	NA	86.2	NA	NA	89.0	NA	NA	91.2
4.2. Regular static immunization services delivered	NA	NA	76.3	NA	NA	84.5	NA	NA	84.8
4.3. Adequate outreach sites	NA	NA	34.5	NA	NA	61.4	NA	NA	68.7
4.4. Catchment area mapped for immunization	NA	NA	32.9	NA	NA	62.2	NA	NA	69.4
4.5. Separate and adequate rooms for immunization services and storing supplies	NA	NA	43.9	NA	NA	53.9	NA	NA	58.4
4.6. All planned outreach sessions were conducted	NA	NA	27.0	NA	NA	52.7	NA	NA	64.3
4.7. Providing health education on immunization	NA	NA	53.8	NA	NA	65.3	NA	NA	71.6
4.8. Immunization services available in all catchment health posts	NA	NA	76.3	NA	NA	86.3	NA	NA	86.1
5. Documentation									
5.1. Electronic database for monitoring immunization and staff training	41.4	30.1	12.5	51.7	47.9	22.2	67.9	40.0	25.8
5.2. Standard operating procedures on immunization	24.1	30.6	23.7	69.0	42.8	43.9	67.9	57.4	56.4
5.3. Reports received from all catchment health posts and documented	NA	NA	67.9	NA	NA	86.4	NA	NA	86.6
5.4. Tally sheets appropriately documented	NA	NA	59.3	NA	NA	57.9	NA	NA	64.8
5.5. Defaulter tracking list	NA	NA	36.7	NA	NA	36.7	NA	NA	83.0
5.6. Standard case definitions for neonatal tetanus, measles, AFP	NA	NA	59.3	NA	NA	59.3	NA	NA	73.3
5.7. Up-to-date under-one register	NA	NA	53.6	NA	NA	53.4	NA	NA	92.5
5.8. Up-to-date national immunization guidelines	62.1	35.2	34.4	86.2	90.7	81.2	89.3	94.2	88.6
6. Coordination and collaboration									
6.1. Established partners’ forum	14.0	NA	NA	20.7	NA	NA	26.4	NA	NA
6.2. Partners’ forum meeting conducted as planned	6.9	NA	NA	6.9	NA	NA	28.6	NA	NA
6.3. EPI programme network	13.8	74.0	NA	93.1	90.2	NA	96.4	94.2	NA
6.4. Established technical working group	NA	30.0	NA	NA	55.4	NA	NA	55.4	NA
6.5. Technical working group meeting conducted in the last quarter	NA	16.8	NA	NA	38.7	NA	NA	43.2	NA
6.6. Conducted catchment area meeting in the last quarter	NA	NA	53.0	NA	NA	83.3	NA	NA	76.2
6.7. Inter-facility linkage on immunization services	NA	NA	83.0	NA	NA	80.0	NA	NA	92.1
7. Monitoring and evaluation									
7.1 Updated EPI monitoring chart posted	62.1	62.8	57.2	79.3	84.9	83.3	89.3	88.0	84.1
7.2. Vaccine logs	NA	NA	48.3	NA	NA	81.9	NA	NA	89.4
7.3. Vaccine and injection materials stock record book	NA	NA	60.3	NA	NA	78.9	NA	NA	89.9
7.4. Conducted internal review meeting on EPI in the last quarter	27.6	35.2	48.3	44.8	64.2	64.8	64.3	71.6	68.9
7.5. SS visit by government or partners in the last quarter	31.0	56.6	65.9	48.3	76.9	72.5	71.4	76.8	79.9
7.6. Data quality check by government or partners in the last quarter	27.6	38.3	34.9	48.3	57.8	64.8	53.6	65.8	70.1
7.7. Timeliness of monthly reports	55.2	55.1	59.8	79.0	80.1	61.9	82.1	94.2	74.0
7.8. Completeness of monthly reports	65.5	61.2	58.1	86.2	89.0	81.9	89.3	94.7	81.5
7.9. Functional performance monitoring team	75.9	60.2	41.4	86.2	84.4	74.2	92.9	90.0	74.0
7.10. Monitored vaccine wastage	41.4	44.9	NA	69.0	55.5	NA	64.3	48.0	NA
7.11. Conducted SS at health posts in the last three months	NA	NA	48.1	NA	NA	43.8	NA	NA	51.6
7.12. Supervised community immunization programme in the last quarter	NA	NA	46.7	NA	NA	64.7	NA	NA	67.8

*Dist*: District health office, *Zone*: Zonal health department; *EPI*: Expanded Programme on Immunization; *HF*: Healthcare facility; *IIP*: Immunization in Practice; *MLM*: Mid-level managers; *REC*: Reaching every community; *SS*: Supportive supervision; *NA*: Not applicable

Regarding planning, only 34.5% of zonal health departments, 45.9% of district health offices, and 27.6% of healthcare facilities had the Reaching Every Community (REC) microplan at baseline to enable them to map community locations and characteristics, and the resources required for routine immunization. After 12 months of technical support, REC microplans were prevalent at 71.4% of zonal health departments, 72.6% of district health offices, and 59.6% of healthcare facilities.

Availability of logistics, supplies, and equipment showed some improvement over time, while items such as refrigerator spare parts were still lacking in zonal health departments 12 months following the interventions (see Table 3). The overall level of logistics and supply at the zonal level showed some improvements, with much better results observed in Oromia and SSNPR.

The baseline analysis for documentation was poor: only 41.4% of zonal health departments, 30.1% of district health offices, and 12.5% of healthcare facilities were able to use the electronic database system for immunization. At the 12th month point of interventions, these figures improved to 67.9, 40, and 25.8%, respectively. Similarly, only 24.1% of zonal health departments, 30.6% of district health offices, and 23.7% of healthcare facilities had standard operating procedures on immunization, which was improved to 67.9, 57.4, and 56.4%, respectively (see Table 3). At baseline, up-to-date national immunization guidelines were available at 62.1% of zonal health departments, 35.2% of district health offices, and 34.4% of healthcare facilities, which was improved to 89.3, 94.2, and 88.6%, respectively, after interventions that included distributing the guidelines from central health bureaus.

In terms of coordination and collaboration, at baseline, 14% of zonal health departments had an established partners’ forum made up of governmental and non-governmental organizations that met every three months and discussed immunization issues in their catchment. Of these, 6.9% were able to conduct a partners’ forum meeting as planned. This improved to 26.4% of zonal health departments having such forums, and 28.6% of these conducting a partners’ forum meeting by the 12th month of interventions. Similarly, 30% of district health offices had an established immunization technical working group, of which 16.8% conducted a technical working group meeting in the last quarter. This was improved to 55.4% of district health offices having such a group, and 43.2% of them conducting a meeting after 12 months of interventions.

Regarding monitoring and evaluation, 62.1% of zonal health departments, 62.8% of district health offices, and 57.2% of healthcare facilities had an updated EPI monitoring chart. After the training and onsite technical support, this improved to 89.3, 88.0, and 84.1%, respectively. Completeness of monthly reports was also improved from 65.5 to 89.3% at the zone level, 61.2 to 94.7% at the district level, and 58.1 to 81.5% at healthcare facility level after interventions. All regions were able to improve their monitoring and evaluation and data management activities, with the improvement significant in SNNPR and Oromia.

Analysing the baseline cumulative score of the six process indicators, the national immunization programme and services capacity was found to be low (< 50%) (see Table 4). Comparing the six parameters within themselves, logistics and supply, as well as human resources had relatively better cumulative scores.

**Table 4:** Baseline percentage scores of process indicators of national immunization programme and service capacity of the six regions

	Process indicator	Mean score (%)		
		Zone (*n* = 29)	District (*n* = 196)	Healthcare facility (*n* = 556)
National immunization capacity represented by six regions	Human resources	56.0	49.9	42.2
	Planning	20.7	31.8	40.5
	Logistics and supply	62.5	54.9	59.1
	Documentation	42.5	33.3	43.7
	Coordination and collaboration	35.3	41.0	68.2
	Monitoring and evaluation	48.3	52.2	50.3

The regional average was computed for baseline process indicators, with Amhara (60.2%) and Afar (51.2%) scoring in the range of 50.0 to 75.0% (fair), while all other regions scoring below 50.0%: Somalia (48.0%), SNNPR (45.5%), Oromia (36.7%), and Gambella (34.9%). There were significant differences in baseline process indicators among regions (*P* < 0.001). Baseline mean values were also compares between developing and emerging regions, with a significant difference found between the two in terms of process indicators at the healthcare facility level (*P* = 0.0001) but not at the district level (*P* = 0.715).

**Fig. 3:**
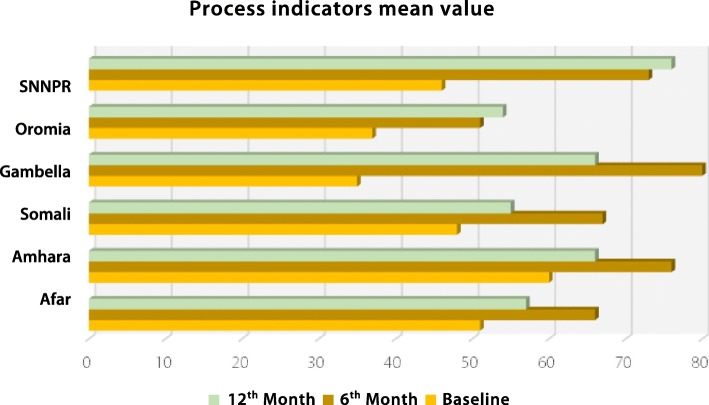
Capacity to deliver immunization program and services at zonal health departments, district health offices, and healthcare facilities from baseline to the 6th and 12th month of interventions in different regions. SNNPR: Southern nations, nationalities and Peoples’ Region

Analysing the cumulative score of the seven process indicators after interventions, the national capacity to deliver immunization programme and services was found to be high, with a score of above 75% which was a major increase from below 50% at baseline; these improvements were observed at the zone, district, and healthcare facility (see Fig. 3) levels. The regional average was computed and it was found that all regions improved their immunization programmes and services after interventions, yet there was a bigger improvement in Gambella and SNNPR both at the 6th and 12th month of interventions as compared to the other regions. Gambella, SNNPR, and Amhara scored ‘good’ at 6th months of interventions and the rest scored ‘fair’. There was a declining trend in some regions from the 6th month to the 12th month of interventions.

### Results of outcome measures

Pentavalent 3, measles, BCG, PCV, and full vaccination status were the outcome measures used to observe changes in immunization coverage. Comparing vaccination coverage from baseline to the 12th month of interventions, it improved significantly from 63.6 to 79.3% for pentavalent (*P* = 0.0001), 62.5 to 72.8% for measles (*P* = 0.009), 62.4 to 73.5% for BCG (*P* = 0.0001), 65.3 to 81.0% for PCV (*P* = 0.02), and insignificantly from 56.2 to 74.2% for full vaccination (see Table 5).

**Table 5:** Percentage scores of outcome measures at baseline, and at the 6th and 12th month of interventions

Outcome measures	Vaccination coverage (% mean)			
	Baseline	At the 6th month	At the 12th month	*P* value
Pentavalent 3	63.57	80.41	79.31	0.0001
Measles	62.46	74.48	72.81	0.009
BCG	62.38	76.69	73.54	0.0001
PCV	65.32	79.87	81.01	0.02
Full vaccination	56.17	88.91	74.20	0.30 ^(not significant)^

*BCG*: Bacillus Calmette–Guérin; *PCV*: Pneumococcal Conjugate Vaccine

**Fig. 4:**
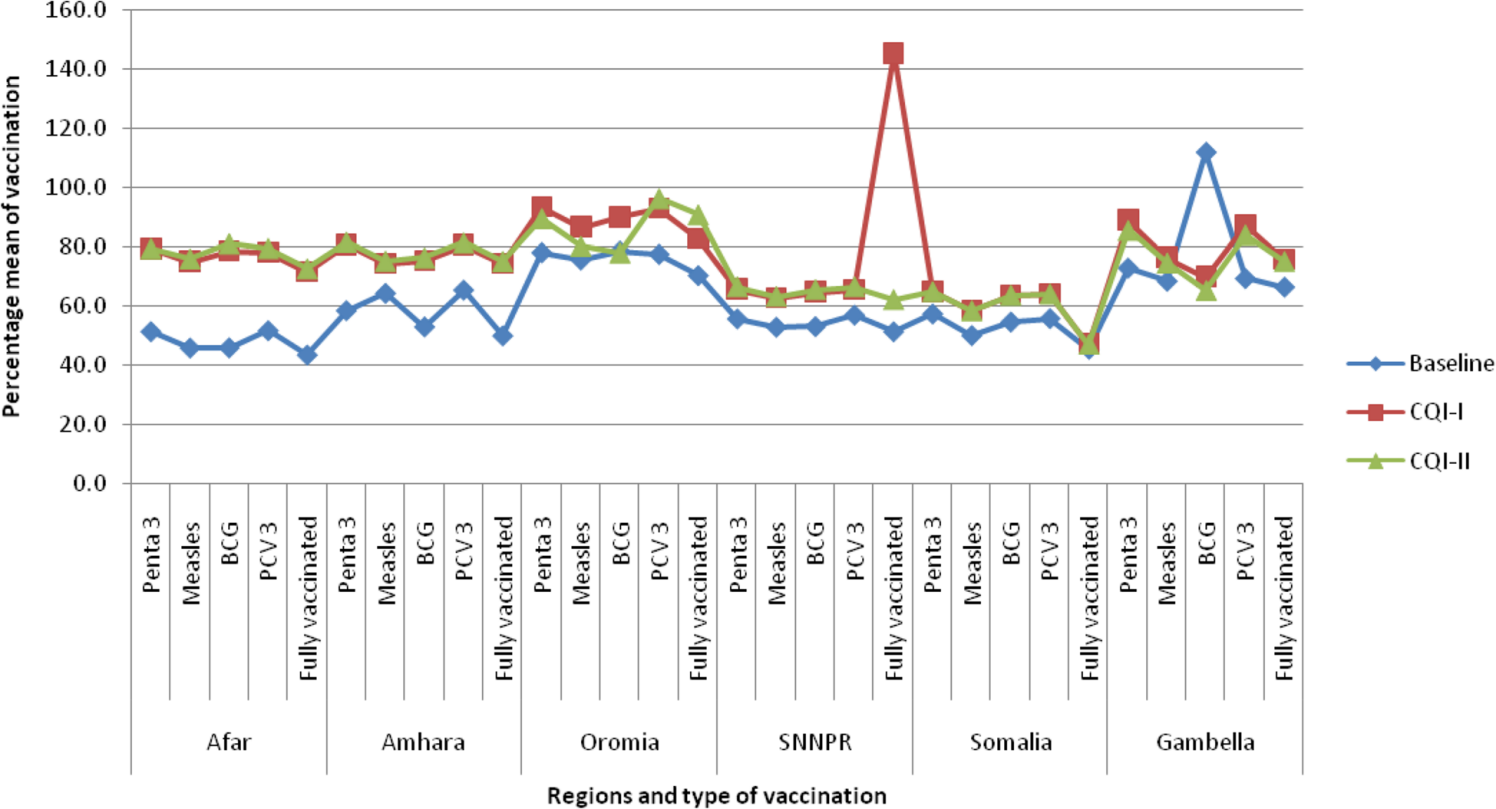
Vaccination coverage from baseline to the 6th and 12th month of interventions in different regions

Tremendous changes were observed in the coverage of all five types of vaccinations in all six regions (see Fig. 4). Full vaccination in SNNPR at the 6th month of interventions and BCG vaccination in Gambella at baseline had exceptionally high values, which could be due to mass vaccination for disease-specific epidemics in these regions. In most regions, an independent *t*-test showed that there were significant changes in the percentage means of all five vaccination types from the baseline to the 12th month of interventions.

## Discussion

The aim of this study was to implement and evaluate the effectiveness of system-wide CQI interventions to improve national immunization programme performance in Ethiopia. The study was able to provide an understanding of the current status of immunization programme and services at public health sectors, thereby producing a consistent immunization interventions platform to monitor, test, and capture changes. The findings revealed significant improvements in process measures and vaccination coverage after 12 months of strengthening local immunization systems.

The success of an immunization programme requires many parameters to be met, among which is knowledge and skills of immunization service providers and peripheral EPI managers [30]. Our study found a critical shortage of qualified experts to handle current and future immunization programmes at the grassroots level. More than 82% of zone and district level health departments and offices lack trained experts to lead and manage immunization programmes. The CQI approach mitigated this by providing MLM training to site EPI experts, empowering them to achieve an optimum standard of delivering the immunization programme in their catchments. A similar approach was followed to channel quality of routine immunization services. Considering the high shortage of healthcare providers to expertly administer immunization services at the healthcare facility and community levels, training on IIP was provided to healthcare workers and health extension workers, enabling them to own and effectively deliver immunization services. Previous studies proved that such training increases knowledge of the frontline staff and boosts overall immunization coverage [31, 32].

At a glance, CQI has a proven track record in improving maternal and new-born health interventions in Tanzania, Uganda, and Ghana [33, 34]; human resources for health in Zambia [35]; and prevention of mother-to-child transmission of HIV in South Africa [36]. In Ethiopia, a few studies have generated evidence on the role of CQI in improving diagnostic capacity at healthcare facilities [37] and strengthening district leadership capacity in support of maternal and neonatal health [38]. This study extended the existing evidence on the realistic use of CQI tailored to Ethiopia’s local context. Beyond this, there is an increasing tendency to devise mechanisms to assure quality of programmes and services in the overall health system in Ethiopia [39–43].

EPI is still the commonly used programmematic approach to reduce morbidity and mortality among children against the most common vaccine-preventable diseases. With this approach, the global vaccine action plan has targeted to prevent millions of deaths by 2020 through more equitable access to vaccines [30]. In agreement with this global target, Ethiopia, through its comprehensive multi-year plan, aims to vaccinate 90% of the unmet population by 2020 [16]. This requires an in-depth understanding of EPI programme and service needs on the ground and the fostering of mechanisms to effectively intervene and sustain promising outcomes. This study proved that knowledge on such national immunization plans is limited in many study sites, and national planning documents to refer to and be guided by were missing. The progress towards effectiveness of national immunization programmes needs to empower regional, zonal, district, and facility-level government health sectors to have accountability and share ownership of the outcomes.

With the study interventions, all regions improved their implementation status, and the gap in immunization between developing and emerging regions narrowed. The CQI approach had the advantage of motivating study sites to maximize their effectiveness, which is an important lesson to transfer to other related health programmes. The existing government structure can responsively host and sustain the approach as part of its routine health programme monitoring and evaluation package.

This study had some limitations. A stronger analysis of the immunization trend could have been obtained if the CQI interventions continued beyond 12 months. Six of the nine regional states were included, thus three were untapped, which could limit the contextualization of findings to the national context. The fact that the study sites represented both developing and emerging regions and that there was a large number of sites are strengths of the study.

## Conclusions

The CQI interventions improved process measures and vaccination coverage in Ethiopia through immunization systems strengthening in a PDCA cycle. The study proved that employing data-driven CQI interventions to assess, improve, and continuously follow up immunization programmes is an effective approach to meet national immunization targets. The approach empowered zone, district, and facility-level government health sectors to exercise accountability and share ownership of immunization outcomes. This approach for improvement of immunization capacity suggests that this model could be adapted to other EPI countries. While universal approaches can improve routine immunization, innovative interventions that target local problems and dynamics are also necessary to achieve optimal coverage.

## Abbreviations

BCG: Bacillus Calmette–Guérin
CQI: Continuous quality improvement
EPI: Expanded Program on Immunization
FMoH: Ethiopian Federal Ministry of Health
HF: Healthcare facility
IEHI: Integrated approach to Empower Healthcare facilities on Immunization
IIP: Immunization in practice
MLM: Mid-level managers
NA: Not available
PCV: Pneumococcal Conjugate Vaccine
PDCA: Plan-Do-Check-Act
REC: Reaching every community
SNNPR: Southern nations, nationalities and Peoples’ Region
SQUIRE 2.0: Standards for Quality Improvement Reporting Excellence
SS: Supportive supervision
WHO: World Health Organization
